# Influenza Virus Aerosols in the Air and Their Infectiousness

**DOI:** 10.1155/2014/859090

**Published:** 2014-08-13

**Authors:** Nikolai Nikitin, Ekaterina Petrova, Ekaterina Trifonova, Olga Karpova

**Affiliations:** Department of Virology, Lomonosov Moscow State University, 1/12 Leninskie Gory, Moscow 119234, Russia

## Abstract

Influenza is one of the most contagious and rapidly spreading infectious diseases and an important global cause of hospital admissions and mortality. There are some amounts of the virus in the air constantly. These amounts is generally not enough to cause disease in people, due to infection prevention by healthy immune systems. However, at a higher concentration of the airborne virus, the risk of human infection increases dramatically. Early detection of the threshold virus concentration is essential for prevention of the spread of influenza infection. This review discusses different approaches for measuring the amount of influenza A virus particles in the air and assessing their infectiousness. Here we also discuss the data describing the relationship between the influenza virus subtypes and virus air transmission, and distribution of viral particles in aerosol drops of different sizes.

## 1. Introduction

Influenza is one of the most contagious and rapidly spreading infectious diseases and an important global cause of hospital admissions and mortality [1]. Influenza virus concentration [2, 3], air circulation time, air temperature, and humidity [4] play an important role in overcoming the epidemic threshold.

Influenza virus particles are constantly circulating in the air (airborne) in different forms (within dust particles or aerosol droplets) [5, 6]. There are some amounts of the virus in the air constantly. These amounts are insufficient to cause disease in people (the immune system of healthy humans prevents infection). However, at a higher concentration of the airborne virus, the risk of human infection increases dramatically.

Early detection of the threshold virus concentration is essential for prevention of the spread of influenza infection. Furthermore, manufacturers are going to integrate detectors of virus particle numbers into hospital air control system equipment. This review discusses different approaches for measuring the amount of influenza A virus particles in the air and assessing their infectiousness.

One of the fundamental works focused on the definition of the harmful concentration of the influenza A virus in the air is a paper by Alford, with coworkers [7]. It is cited in many recent reports [8–10]. A study was initiated to determine the minimum infectious aerosol dose and the resulting patterns of infection and illness. Observations made during experimental infections with human volunteers are particularly interesting and relevant. In studies conducted by Alford and colleagues [7], volunteers were exposed to carefully titrated aerosolized influenza virus suspensions by inhaling through a face mask. The demonstration of infection in participants of the study was achieved by recovery of infectious viruses from throat swabs, taken daily, or by seroconversion, that is, the development of neutralizing antibodies. The use of carefully titrated viral stocks enabled the determination of the minimal infectious dose by aerosol inoculation. The approximate 50% human infectious dose (HID_50_-50% human infectious dose) of virus per volunteer was from 1 to 126 TCID_50_ (the tissue culture 50% infectious dose). The dose for half of the volunteers was 5 TCID_50_. The other half of the men, who had very low or nondetectable preinoculation antibody titers, were infected with 0.6 to 3 TCID_50_. The study reliably shows that the human infectious dose of the influenza A virus, when administered by aerosol to subjects free of serum neutralizing antibodies, is approximately 3 TCID_50_. The approaches used in this study allow the precise number of infectious particles in the total number of particles to be determined.

Ward, with coworkers [11], confirmed experimentally that three log_10_ copies/mL corresponded to 1 TCID_50_/mL. That is, one TCID_50_/mL contains 1000 copies of the viral genome.

According to other reports, the aerosol infection dose for humans was about 1.95 × 10^3^ viral genome copies, for approximately 300–650 copies of human influenza viruses were contained in 1 TCID_50,_ according to previous studies [9, 12].

During the 2009-2010 influenza season (from December to April), Yang, with coworkers [10], collected samples from a health centre, a day-care centre, and airplanes. The concentrations of airborne influenza viruses (A/PR/8/34 (H1N1) and A/swine/Minnesota/1145/2007 (H3N2)) were measured. The influenza A virus RNA was quantified by RT-PCR. Fifty percent of the samples collected contained the influenza A virus, with concentrations ranging from 5.8 × 10^3^ to 3.7 × 10^4^ genome copies per m^3^. The average concentration of the virus was 1.6 ± 0.9 × 10^4^ genome copies per m^3^, corresponding to 35.4 ± 21.0 TCID_50_ per m^3^ air. According to Yang et al. [10], 1 TCID_50_ of A/PR/8/34 (H1N1) stock was equivalent to 2.1 × 10^3^ genome copies, and the ratio for the pandemic A/California/04/2009 (H1N1) strain was determined to be 452 ± 84 genome copies per TCID_50_.

Using the measured airborne virus concentration and an adult breathing rate, Yang, with colleagues [10], estimated the inhalation doses during exposures of 1 h (e.g., the duration of a clinical visit), 8 h (a workday), and 24 h to be 1.35 × 10^4^, 1.06 × 10^5^, and 3.2 × 10^6^ viral particles (or 30 ± 18, 236 ± 140, and 708 ± 419 TCID_50_), respectively. Compared with the aerosol HID_50_ 0.6–3 TCID_50_ [7], these doses are adequate to induce infection. In other words, over 1 h, the inhalation dose is estimated to be 30 ± 18 TCID_50_ or about 16 000 particles of the influenza A virus, which is more than enough to induce infection.

## 2. RT-PCR is the Principal Method for Virus Particle Determination in the Air

To determine the concentration of virus particles in the air, a quantitative reverse transcription polymerase chain reaction (RT-PCR) method is often used [2, 10, 13–15]. Some detection limits for the influenza A virus matrix gene reported recently by PCR are 0.1 TCID_50_/mL [16], 0.2 TCID_50_/mL [17], and 0.006–0.02 TCID_50_/mL [12] or 0.01–0.1 TCID_50_/mL by LightCycler [18]. In some studies a difference in sensitivity of RT-PCR for different subtypes of the influenza A virus was observed. The RT-PCR showed sensitivity of 350 copies of H3N2 and 120 copies of H1N1 per reaction, representing the influenza A types in common circulation at the time of the study [19]. In another study [20], the influenza virus subtypes H1 and H3 have been successfully identified with equal efficiency.

However, this method does not always provide an adequate result. RT-PCR allows for the obtaining of information on the total number of viral particles, but not on the number of infectious particles. Simply testing aerosols by RT-PCR for detection of viral nucleic acid would not be sufficient to demonstrate that the viruses in fine particles remain infectious. Given the extensive debate in the literature [21, 22] and the likelihood that a large percentage of viral copies detected by molecular methods are defective [23, 24], it would be important for new studies to quantify infectious viruses and not merely measure the total viral RNA copy numbers. Based on RT-PCR assay and the influenza virus stock used for calibration, Fabian, with colleagues [9], established a ratio of 300 copies per TCID_50_, which is well within the previously published estimates of 100–350 or 650 [12, 25].

## 3. Distribution of Viral Particles in the Aerosol Depending on the Size of the Drops

Alford and colleagues [7] studied the aerosol particles of influenza virus suspensions with a diameter of 1–3 *μ*m. Blachere, with colleagues [26], revealed that 46% of influenza virus particles were found in the first stage of the samplers, which collected particles with a diameter of >4 *μ*m. However, 49% of the isolates were collected in the second stage, which collects particles with a diameter of 1–4 *μ*m, and 4% were collected on the back-up filter, which collects particles with a diameter of <1 *μ*m. These findings indicate that 99% of the total viral particles were found in the respiratory aerosol fraction. Coughing, sneezing, talking, and breathing generate a cloud of airborne particles with diameters that can range from a few millimeters to <1 *μ*m [27–30]. Large droplets (>50 *μ*m in diameter) settle on the ground almost immediately, and intermediate-sized droplets (10–50 *μ*m) settle within several minutes. Small particles (<10 *μ*m), including droplet nuclei from evaporated larger particles, can remain airborne for hours and are easily inhaled deep into the respiratory tract. Fabian, with coworkers [9], detected the influenza virus RNA in the exhaled breath of patients and found that >99% of exhaled particles were <5.0 *μ*m in diameter. These findings regarding the influenza virus RNA suggest that the influenza virus may be contained in fine particles generated during tidal breathing and add to the body of literature suggesting that fine particle aerosols may play a role in influenza transmission. Calculation of Stokes' law on settling rate indicated that it took 67 min for a particle with an aerodynamic diameter of 5 *μ*m to settle down from a 3 m height in the static environment; and particles of ≤5 *μ*m could reach as far as pulmonary alveoli [6]. Lindsley et al. [13] found that a 4-*μ*m particle takes 33 min to settle 1 m in still air, and a 1-*μ*m particle takes 8 h; in addition, room air mixing and turbulence can keep these particles airborne even longer. Bischoff, with colleagues [31], later clarified that up to 89% of influenza virus-carrying particles were <4.7 *μ*m in diameter. Other works confirming this data were carried out on different subtypes of the influenza A virus [2, 13, 14].

Infectious viruses and viral RNA can be detected in both larger particles of >5 *μ*m and smaller particles of <5 *μ*m [9, 14, 32]. Experimental studies have demonstrated that the influenza virus can remain infectious in small particle aerosols and can transit across rooms [24, 33]. Cowling et al. [33] found that aerosol transmission (particles <5 *μ*m) accounted for approximately half of all transmission events. Infectious influenza was recovered in all aerosol fractions (5.0% in >4 *μ*m aerodynamic diameter, 75.5% in 1–4 *μ*m, and 19.5% in <1 *μ*m; *n* = 5) [24].

The aerosol fraction that is <4 *μ*m (the “respirable fraction”) is of particular concern because it can remain airborne for an extended time and disperse throughout a room occupied by a patient with influenza. Also, particles containing influenza RNA are small enough to be drawn down into the alveolar region of the lungs. The infectious dose required for inoculation by the aerosol route relative to contact or droplet transmission is unclear, but two reviews of previous studies concluded that the infectious dose by the aerosol route is likely to be considerably lower than the infectious dose by intranasal inoculation [21, 34] and that aerosol inoculation results in more severe symptoms [21], presumably because aerosol particles are able to deposit deeper in the respiratory tract. However, the viability of influenza viruses in particles of different sizes and the persistence of viable airborne viruses in the environment are not yet known.

## 4. Viability and Infectivity of Airborne Influenza Virus Particles Depend on Environmental Conditions

Numerous reports have shown that the viability of different airborne viruses is dependent on environmental conditions and on the methods of collection and handling of bioaerosol samples [35]. For example, the survival of airborne influenza was shown to greatly depend on the relative humidity (RH), as well as on ambient air temperature and ultraviolet radiation levels [34].

The infectivity of influenza virus particles is preserved depending on temperature, pH and salinity of the water, and UV irradiation. At 4°C, the half-life of infectivity is about 2-3 weeks in water. Due to the conformation of the lipid bilayer, survival under normal environmental conditions should be shorter. Infectivity of the influenza virus particle is easily inactivated by all alcoholic disinfectants, chlorine and aldehydes. As far as is known, temperatures above 70°C destroy infectivity in a few seconds [36].

Using the newly developed guinea pig model of the influenza virus transmission, Lowen and coauthors [37] tested the impact of ambient temperature and relative humidity (RH) on the efficiency of viral spread between hosts. When inoculated and exposed guinea pigs were housed in separate cages, transmission was found to be dependent on both temperature and RH [37–39]. Among the temperatures tested, transmission was highly efficient at 5°C but was blocked or inefficient at 30°C. Dry conditions (20% and 35% RH) were also found to be more favourable for spread than either intermediate (50% RH) or humid (80% RH) conditions. These identical results were obtained using a seasonal human strain, A/Panama/2007/1999 (H3N2) and A/Netherlands/602/2009 (H1N1). Yang and coauthors [40] propose that the effect of RH on virus viability is mediated by salt concentration within droplets: at high RH, physiological concentrations are maintained and viruses are relatively stable; at intermediate RH, evaporation leads to increased salt concentration, resulting in virus inactivation; and at low RH (<50%), salts crystallize out of solution, yielding low salt concentrations and high virion stability. Pica, with colleagues [41], tested two influenza B viruses transmission at low (5°C) versus intermediate (20°C) temperatures. The transmission was more efficient under colder conditions. Thus, transmission of human influenza viruses by a respiratory droplet or aerosol route in the guinea pig model proceeds most readily under cold, dry conditions. These findings suggested two means by which environmental factors could drive the wintertime seasonality of influenza.

Atkinson and Wein [8] created a mathematical model that describes aerosol (i.e. droplet-nuclei) and contact transmission of influenza A virus subtype H5N1 within a household containing one infected. It was demonstrated that in addition to the concentration of particles in the air that a person inhales, time plays a determining role in the influenza virus infection.

## 5. Relationship between the Influenza Virus Subtypes and Virus Air Transmission

Is there any difference in the influenza virus transmission depending on the virus subtype? In scientific publications, contradictory data obtained on laboratory animals only are presented. Studies using the guinea pig and the ferret models have demonstrated differences of the influenza virus transmission for different strains or genetic compositions by the aerosol route [37, 38, 42].

In the study by Chou, with coworkers [43], the aerosol transmission rate of an influenza virus A/California/04/09 (H1N1) and another H1N1 strain, A/Puerto Rico/8/34, was measured as the percentage of susceptible guinea pigs infected following exposure to inoculated animals. A/California/04/09 was found to spread more efficiently. Differences in the nucleotide sequence of the M segment of the virus genome were found to cause a difference in the aerosol transmission rate. Interestingly, the Eurasian avian-like swine viruses, which possess an M segment closely related to that of the A/California/04/09 (H1N1) virus, are not transmitted efficiently in humans [44].

Pearce, with colleagues [45], characterized four A(H3N2)v viruses isolated in 2009, 2010, and 2011, from patients with uncomplicated upper respiratory tract illnesses (A/Kansas/13/2009 (KS/09), A/Minnesota/11/10 (MN/10), A/Pennsylvania/14/, 10 (PA/10), and A/Indiana/08/11 (IN/11)) and demonstrated that the 2010-2011 A(H3N2)v viruses replicated efficiently in ferrets and readily transmitted in both the direct-contact (DC) and respiratory-droplet (RD) models, whereas the 2009 A(H3N2)v virus exhibited efficient DC transmission but less efficient RD transmission. Typically, the difference in efficiency of the infection of animals in all experiments was a delay in infection of 0.5–1 day. In this context, it is difficult to claim that a clear relationship between the influenza virus subtypes and air virus transmission can be revealed. Authors do not exclude that other genetic requirements must be met in order for the transfer to take place.

The study by Chan, with colleagues [46], has demonstrated that the sensitivity of the commercially available rapid influenza antigen detection tests did not depend on the subtype influenza virus. The analytical sensitivity of the detection tests for swine influenza virus (TCID_50_ log_10_ 3.3–4.7) was comparable with that of seasonal influenza A/HK/403946/09 (H1N1) virus (TCID_50_ log_10_ 4.0–4.5). Thus, differences between subtypes were not identified.

Clear correlation and dependence of the number of diseased subjects on the concentration of the influenza virus in the air were shown in various models. For example, a relationship between the number of infected pigs and the influenza detection in the air was identified in a study on a single H1N1 viral strain [47]. The chance of detection of an influenza positive air sample increased 2.2 times per each additional nasal secretion by a sick pig. This suggests that the risk of aerosolization and perhaps aerosol transmission increases as the number of infected pigs increase.

## 6. Exhaled Breath of Healthy Subjects also Contains Influenza Virus Particles

It is important to consider that the air exhaled by the healthy person also contains influenza virus particles. In studies of particles exhaled by healthy subjects during tidal breathing, researchers reported concentrations from 1 to over 1 × 10^4^ particles per liter, with the majority of the particles being less than 0.3 *μ*m in diameter [29, 48, 49]. One of these studies also reported that 55% of the population studied exhaled >98% of the particles in the air volume investigated and concluded that these subjects, classified as high producers, could, over time, exhale more particles during normal tidal breathing than during relatively infrequent coughing or sneezing events [49]. Concentrations in exhaled breath samples ranged from <48 to 300 influenza virus RNA copies per filter in the positive samples, corresponding to exhaled breath generation rates ranging from <3.2 to 20 influenza virus RNA copies per minute. Total particle concentrations ranged from 67 to 8.5 × 10^3^ particles per liter of air.

## 7. Conclusions

The human infectious dose of the influenza A virus, when administered by aerosol to subjects free of serum neutralizing antibodies, ranges between 1.95 × 10^3^ and 3.0 × 10^3^ viral particles.

To determine the concentration of virus particles in the air, the RT-PCR method is often used. However, RT-PCR analysis provides information on the total number of viral particles, but not on the number of infectious particles. Influenza virus genomic segments are chosen and packaged at random, whereby only parts of the virions are infectious.

According to various scientific publications, data about the influence of the virus subtype on the effectiveness of influenza transmission are contradictory. The subtype-specific differences in influenza virus transmission were observed in animal models, and recipient animals did not exhibit a preexisting influenza virus specific immune response. However, the pathogenicity of a virus subtype depends on the immune status of the recipients (human). The second point is (when) how recently viruses of the same subtype circulated in the population previously.

Therefore, it is important to consider that the risk of acquiring influenza is determined by both the concentration of the influenza A virus infectious particles (not their total amount) in the air and the immune status of the exposed individuals.
