# Corrigendum to “Influenza Virus Aerosols in the Air and Their Infectiousness”

**DOI:** 10.1155/2024/9762961

**Published:** 2024-07-23

**Authors:** Nikolai Nikitin, Ekaterina Petrova, Ekaterina Trifonova, Olga Karpova

**Affiliations:** Department of Virology Lomonosov Moscow State University, 1/12 Leninskie Gory, Moscow 119234, Russia

In the article titled “Influenza Virus Aerosols in the Air and Their Infectiousness” [1], concerns were raised regarding the lack of supporting evidence for the suggestion that air exhaled by a healthy person also contains influenza virus particles. The authors found that there were errors in Section 6, introduced during revisions. They apologise for the errors and Section 6 should now read as below:

6. Influenza Virus and Aerosol Particles in Human Exhaled Breath

In studies of particles exhaled by healthy subjects during tidal breathing, researchers reported concentrations from 1 to over 1 × 10^4^ particles per liter, with the majority of the particles being less than 0.3  *μ*m in diameter [29, 48, 49]. One of these studies also reported that 55% of the population studied exhaled >98% of the particles in the air volume investigated and concluded that these subjects, classified as high producers, could, over time, exhale more particles during normal tidal breathing than during relatively infrequent coughing or sneezing events [49]. Concentrations in exhaled breath of influenza-infected patient samples ranged from <48 to 300 influenza virus RNA copies per filter in the positive samples, corresponding to exhaled breath generation rates ranging from <3.2 to 20 influenza virus RNA copies per minute. Total particle concentrations ranged from 67 to 8.5 × 10^3^ particles per liter of air [9].

These changes do not affect the conclusions of this article.
